# The stem-like Stat3-responsive cells of zebrafish intestine are Wnt/β-catenin dependent

**DOI:** 10.1242/dev.188987

**Published:** 2020-06-19

**Authors:** Margherita Peron, Alberto Dinarello, Giacomo Meneghetti, Laura Martorano, Nicola Facchinello, Andrea Vettori, Giorgio Licciardello, Natascia Tiso, Francesco Argenton

**Affiliations:** Dipartimento di Biologia, Università degli Studi di Padova, Via Ugo Bassi 58b, 35121 Padova, Italy

**Keywords:** Stat3, Stem cells, Intestine, Zebrafish, Wnt/β-catenin

## Abstract

The transcription factor Stat3 is required for proliferation and pluripotency of embryonic stem cells; we have prepared and characterized fluorescent Stat3-reporter zebrafish based on repeats of minimal responsive elements. These transgenic lines mimic *in vivo* Stat3 expression patterns and are responsive to exogenous Stat3; notably, fluorescence is inhibited by both *stat3* knockout and IL6/Jak/STAT inhibitors. At larval stages, Stat3 reporter activity correlates with proliferating regions of the brain, haematopoietic tissue and intestine. In the adult gut, the reporter is active in sparse proliferating cells, located at the base of intestinal folds, expressing the stemness marker *sox9b* and having the morphology of mammalian crypt base columnar cells; noteworthy, zebrafish *stat3* mutants show defects in intestinal folding. Stat3 reporter activity in the gut is abolished with mutation of T cell factor 4 (Tcf7l2), the intestinal mediator of Wnt/β-catenin-dependent transcription. The Wnt/β-catenin dependence of Stat3 activity in the gut is confirmed by abrupt expansion of Stat3-positive cells in intestinal adenomas of *apc* heterozygotes. Our findings indicate that Jak/Stat3 signalling is needed for intestinal stem cell maintenance and possibly crucial in controlling Wnt/β-catenin-dependent colorectal cancer cell proliferation.

## Abstract (precis)

**Summary:** Using a fluorescent reporter for Stat3 activity, we have identified the stem cells of zebrafish intestine and characterized their Wnt requirements and responsiveness.

## INTRODUCTION

Signal transducer and activator of transcription 3 (Stat3), the most prominent member of the STAT family of proteins, is involved in a plethora of cellular processes including development, differentiation, inflammation and metabolism (Levy and Lee, 2002). In mammals, Stat3 is typically activated by janus kinase (Jak) 2-mediated phosphorylation of its tyrosine at position 705. Once phosphorylated at Y705, Stat3 dimerizes, translocates in the nucleus and binds to Stat3 binding elements (SBEs) in the regulatory regions of its target genes.

One of the most remarkable functions of Stat3 is its role in preserving the full differentiative potential of stem cells in several tissues. Indeed, Stat3 is required for self-renewal of mouse embryonic stem cells (Raz et al., 1999), survival of murine small-intestine crypts (Matthews et al., 2011), maintenance of neural precursors as well as for regulation of haematopoiesis and muscle regeneration in adult mice (Galoczova et al., 2018). Although Stat3 phosphorylation is strictly regulated in physiological conditions, over-activation of Stat3 has been detected in a variety of human cancers, suggesting a key role for this transcription factor in tumour initiation and progression (Huynh et al., 2019). In haematopoietic malignancies and solid tumours, the chronic inflammatory conditions and the maintenance of cancer stem cell properties are reported to be dependent on the IL-6/Stat3 axis, which also stimulates the self-renewal of neoplastic cells (Pattabiraman and Weinberg, 2014; Johnson et al., 2018). In particular, the importance of Jak/Stat3 signalling in intestinal tumorigenesis is well established and associated with the hyperproliferative and invasive phenotype of human colorectal cancer (CRC) (Wang and Sun, 2014). Although IL-6/Stat3 signalling alters the mucosal barrier in colon adenomas, accelerating the adenocarcinomas transition, it is also well known that the Wnt/β-catenin pathway plays an essential role in gut homeostasis (Ahmad et al., 2017). In fact, it has been reported that hyperactivated Wnt/β-catenin signalling induces epithelial to mesenchymal transition and promotes CRC (Morin et al., 1997; Vu and Datta, 2017), indicating that the two signalling pathways both act in CRC pathogenesis and progression (Ahmad et al., 2017).

Germline transformed zebrafish embryos expressing fluorescent protein-coding genes under the control of specific promoters and enhancers can be used to follow gene regulation in specific cell populations at single-cell resolution. These transgenic animals, either embryos, larvae or adults, can be studied with digital time-lapse image quantification, fluorescence-activated cell sorting (FACS) and mass sequencing at specific times and in specific tissues. In addition, global fluorescence can be used as an output for screening new compounds as well as for drug repurposing (Gallardo and Behra, 2013). This approach is largely used to study entire promoters but, lately, has been improved by utilising multimerized elements that respond to single transcription factors (Moro et al., 2013). *In vitro* studies have shown that the TTCCCGAA sequence taken from the C-reactive protein (CRP) promoter (CRP acute phase response element CRP-APRE) is able to selectively mediate Stat3 transcriptional activity in response to IL-6 in Hep3B cells (Zhang et al., 1996). As a consequence, multimerized CRP-APRE has been widely used as a bona-fide Stat3-specific reporter in mammalian cells (Turkson et al., 1998). Taking advantage of zebrafish reporter lines as living biosensors and the specificity of CRP-APRE as a Stat3-responsive element, we generated a Stat3 transgenic reporter to clarify *in vivo* the role of this transcription factor during zebrafish embryonic and larval development, in zebrafish adults and in intestinal cancer models. Our analyses reveal that Stat3 activity is tightly linked with proliferation, that Stat3-responsive cells of zebrafish intestinal folds co-localize with the stemness marker Sox9b (Aghaallaei et al., 2016), and that gut cellular activities are dependent on a Wnt/β-catenin/Stat3 signalling cascade, both during tissue formation and tumour growth.

## RESULTS

### The zebrafish *Tg(7xStat3-Hsv.Ul23:EGFP)* fluorescent line reveals regions of Stat3 transcriptional activity

**Fig. 1.**
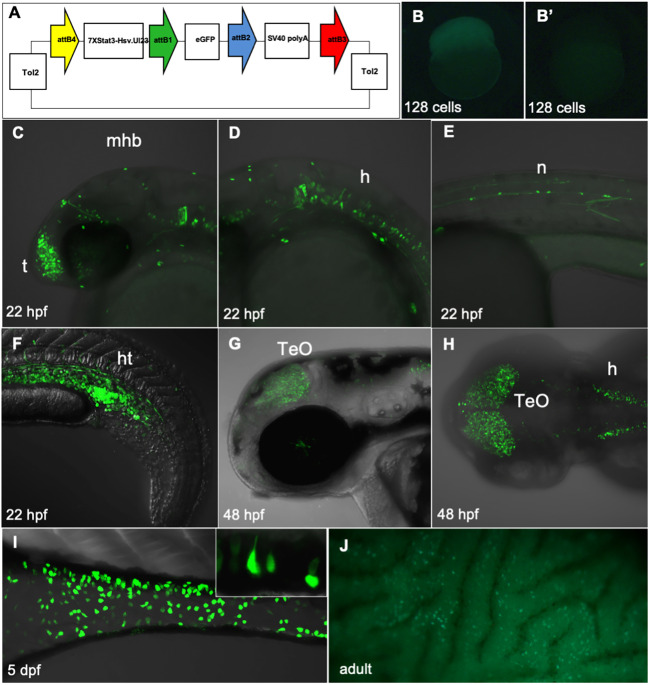
**Generation of *Tg(7xStat3-Hsv.Ul23:EGFP)* fish and characterization of their expression pattern.** (A) Scheme of the Tol-2 vectors used to generate the *Tg(7xStat3-Hsv.Ul23:EGFP)* reporter. (B,B′) Diffused EGFP is detectable in early stage embryos obtained by outcrossing transgenic females (B), not with transgenic males (B′). (C-F) At 22 hpf, EGFP expression is detectable in the anterior telencephalic region (t), the primordial midbrain hindbrain boundary (mhb), the hindbrain (h), the primitive neuromasts (n) and in the haematopoietic tissue (ht). (G,H) At 48 hpf, EGFP expression is mostly located in the optic tectum (TeO) and in the hindbrain (h). (I,J) Starting from 4 dpf, EGFP expression is detectable in the developing intestine in isolated pear-shaped cells (I); the intestinal fluorescence lasts throughout adulthood (J).

With the aim of detecting Stat3-responsive cells in developing zebrafish embryos, we generated a zebrafish transgenic line in which tandemly repeated Stat3-responsive elements were used to drive a fluorescent reporter (Moro et al., 2012, 2013). To this purpose, seven repeats containing the Stat3-responsive element (TTCCCGAA) from the human CRP-APRE were taken from a pLucTKS3 construct (Turkson et al., 1998), cloned upstream of a 24-bp fragment of the herpes simplex virus thymidine kinase (Hsv.Ul23) promoter and used to create the Tol2 Gateway destination vector *pDest(7xStat3-Hsv.Ul23:EGFP)* (Fig. 1A) (Kwan et al., 2007). One-cell stage embryos injected with the destination vector were raised to adulthood and outcrossed with wild-type fish in order to isolate founders carrying the transgenic cassette in the germ-line. F_1_ progenies from single founders, selected on the basis of their Mendelian transmission and reporter signal intensity, were raised to adulthood to establish the stable and hemizygous transgenic line *Tg(7xStat3-Hsv.Ul23:EGFP)* [called *Tg(7XStat3:EGFP)* from now on].

The reporter expression was already detectable shortly after fertilization: when eggs were laid by a *Tg(7xStat3:EGFP)* female, embryos displayed ubiquitous EGFP expression even at the one-cell stage. On the other hand, no EGFP was detectable in one-cell stage embryos obtained by outcrossing *Tg(7xStat3:EGFP)* males with wild-type females, thus indicating that the reporter was maternally activated in the oocyte during oogenesis (Fig. 1B,B′). High levels of zygotic transgene expression start to be detected during late somitogenesis (22 hpf, hours post fertilization) in the anterior telencephalon (Fig. 1C), in the primordium of the midbrain–hindbrain boundary (MHB) and in cells of the hindbrain region that possibly define the precursors of zebrafish cranial ganglia (Fig. 1D). In addition, at the same stage of development, *Tg(7xStat3:EGFP)* transgene expression could be detected in neuromast precursor cells of the head and lateral line (Fig. 1E). Furthermore, the developing haematopoietic tissue, that in zebrafish is topologically identified posteriorly to the yolk extension in the intermediate cell mass (ICM), revealed a strong transgene activity detectable from 19-20 hpf (Fig. 1F), consistent with the known activity of Stat3 during mammalian haematopoietic stem cell regeneration (Chung et al., 2006). This was also confirmed by crossing the *Tg(7xStat3:EGFP)* line with the *Tg(gata1a:DsRed)^sd2^* line that labels erythroid progenitor cells (Traver et al., 2003); at 22 hpf the two fluorescent proteins were partially co-expressed in cells of the haematopoietic tissue (Fig. S1). At 48 hpf, *Tg(7xStat3:EGFP)* activity was mostly located in cells of the optic tectum (TeO), a tissue known to be rapidly proliferating and differentiating at this stage (Fig. 1G,H) (Recher et al., 2013). EGFP expression in TeO was inversely proportional to the differentiation gradient, being stronger in the peripheral midbrain layer (PML) of the TeO and then progressively fading at the centre of the lobe (Fig. 1H). More detailed information concerning Stat3 reporter expression in the CNS has been obtained using VIBE-Z imaging (Ronneberger et al., 2012) of larvae at 3 dpf (days post fertilization). At this stage, *Tg(7xStat3:EGFP)* activity was also found in the forebrain (*subpallium*, preoptic region and hypothalamus) and the retinal layer (Fig. S2, Movie 1). Starting from 4 dpf, a strong EGFP expression was detected in a restricted population of pear-shaped cells of the developing intestine (Fig. 1I); notably, this ‘salt and pepper’ EGFP fluorescence was maintained in the adult intestine (Fig. 1J).

**Fig. 2.**
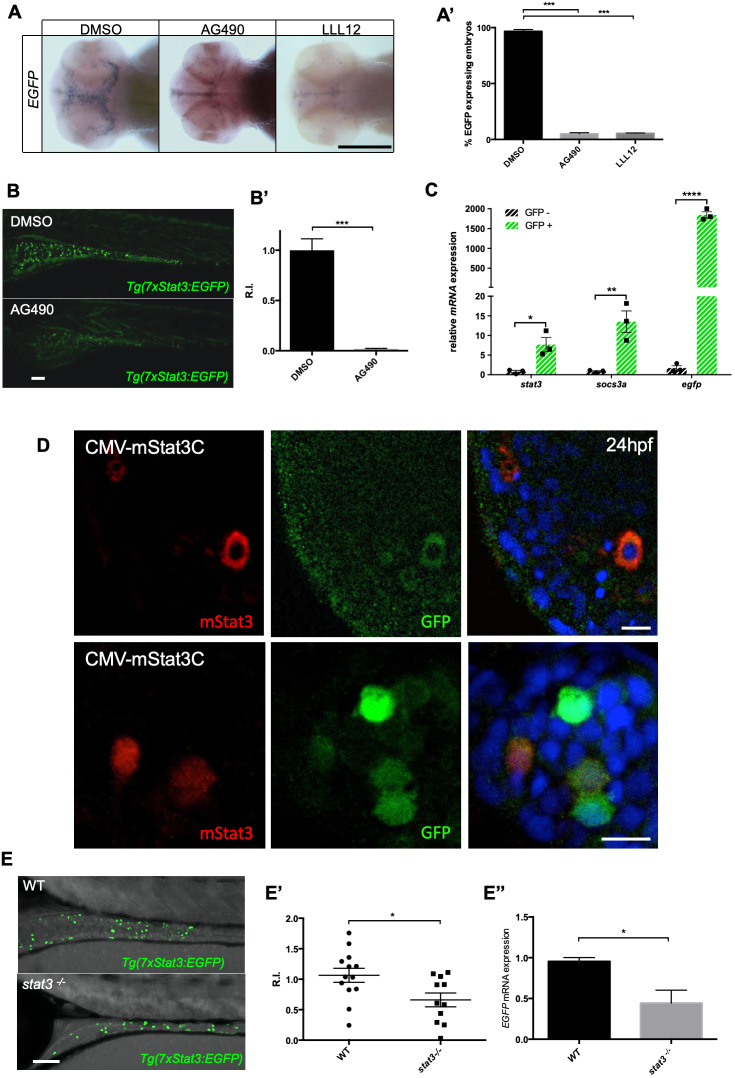
***Tg(7xStat3:EGFP)* is a bona fide Stat3 pathway reporter.** (A) Whole mount *in situ* hybridization detection of *EGFP* mRNA in the optic tectum of 48 hpf embryos treated with inhibitors of the Stat3 pathway, AG-490 and LLL12; control embryos were treated with DMSO. (A′) Percentage of samples displaying *EGFP* mRNA expression in the optic tectum (three independent biological samples; *n*=40). (B) Fluorescent image of *Tg(7xStat3:EGFP)* intestine at 6 dpf after 3 days of either 60 µM AG-490 or DMSO treatment. (B′) EGFP fluorescence quantification in the intestine of AG-490 and control larvae after 3 days of 60 µM AG-490 administration (*n*=20). (C) qRT-PCR analysis of *stat3*, *socs3a* and *EGFP* expression in EGFP-positive and EGFP-negative cells taken from adult intestines. (D) Immunofluorescence against mouse Stat3 (red spots) and colocalization with EGFP fluorescence of *Tg(7xStat3:EGFP)* larvae injected with CMV-m*Stat3C* plasmid. (E) Live image of the intestine of 7 dpf *stat3^−/−^/Tg(7xStat3:EGFP)* and *stat3^+/+^/Tg(7xStat3:EGFP)* siblings. (E′) EGFP fluorescence quantification in the intestine of wild-type (WT) and *stat3^−/−^ Tg(7xStat3:EGFP)* larvae at 7 dpf (*n*=12, *P*=0.0211). (E″) qRT-PCR analysis of *EGFP* expression in 7 dpf *stat3^−/−^/Tg(7xStat3:EGFP)* larvae with respect to the *stat3^+/+^/Tg(7xStat3:EGFP)* sibling (three biological samples). All statistical analyses were performed by unpaired *t*-test; *P<0.05, **P<0.01, ***P<0.001, ****P<0.0001. Graphs indicate mean±s.e.m. Scale bars: 100 μm (A,B,E), 10 μm (D).

Noteworthy, Stat3 reporter fluorescence reflects *stat3* mRNA expression domains at all stages of zebrafish embryonic development, including telencephalon, retina, cranial ganglia, lateral line system and TeO, as already described by Thisse et al. (2004). To further assess Stat3 reporter responsiveness, we provided the embryos with two known chemical inhibitors of the pathway: AG-490, a known Jak2 kinase inhibitor, and LLL12, a molecular competitor that binds specifically to the dimerization binding region of the Stat3 protein, thus preventing its nuclear translocation (Lin et al., 2010; Meydan et al., 1996). Both AG-490 and LLL12 treatments resulted in significant reduction of EGFP expression in the TeO compared with control (Fig. 2A,A′); moreover, AG-490 treatment between 3 and 6 dpf almost completely abolished EGFP intestinal expression in *Tg(7xStat3:EGFP)* larvae (Fig. 2B,B′).

To test their positive responsiveness to Stat3, we injected one-cell stage *Tg(7xStat3:EGFP)* embryos with an mRNA encoding a constitutively active form of murine Stat3 harbouring cysteine substitutions at residues 661 and 663 (m*Stat3C*). These modifications force the formation of disulphide bridges and consequent induction of a constitutively active Stat3 dimer (Sellier et al., 2013). Embryos injected with m*Stat3C* mRNA displayed an ectopic and significant increase in EGFP fluorescence (Fig. S3A,A′), confirming that *Tg(7xStat3:EGFP)* reporter responsiveness *in vivo* is bona-fide Stat3 dependent. These data were also confirmed by immunofluorescence studies performed in *Tg(7xStat3:EGFP)* larvae injected with CMV-m*Stat3C* plasmid, which showed mosaic co-localization of EGFP ectopic signal and mouse Stat3 protein (Fig. 2D). In addition, we generated the CRISPR/Cas9 *stat3^ia23/ia23^* knockout line (from now on named *stat3^−/−^*). These *stat3^−/−^* fish are predicted to encode a truncated protein of 456 amino acids, thus lacking all functional domains, including the DNA-binding domain, the dimerization domain and the transactivation domain (Fig. S4A). At 6 dpf, *stat3* mutant larvae show both a significant reduction in mutant RNA expression (Fig. S4B), consistent with nonsense RNA decay and confirming the null nature of this allele, and a significant decrease in the transcription of two known Stat3 target genes, *socs3a* and *cebpb* (Fig. S4C). Notably, *Tg(7xStat3:EGFP)* reporter larvae in *stat3^−/−^* background display a significant reduction in intestinal EGFP fluorescence at 5 dpf with respect to wild-type siblings (Fig. 2E,E′), together with a corresponding 50% reduction in *EGFP* transcripts (Fig. 2E″). Moreover, to overcome compensation effects present in a genetic mutant and the maternal inherited mRNAs, we injected a previously validated translation blocking morpholino (stat3-MO-1) in the *Tg(7xStat3:EGFP)* reporter, using a five-base mismatch morpholino (5mism-MO) as control (Miyagi et al., 2004; Liu et al., 2017; Yamashita et al., 2002). The silencing led to a reduction in reporter fluorescence to 25% with respect to fish injected with 5mism-MO at 24 hpf and 48 hpf (Fig. S3B,B′,C,C′); this is consistent with the data obtained with the mutant line at later stages (Fig. 2D).

In addition, we performed FACS on dissociated adult intestine and isolated EGFP-positive and EGFP-negative cells. Then, we performed quantitative real-time reverse transcription PCR (qRT-PCR) to analyse the mRNA levels of *stat3*, *socs3a* and *EGFP* (Fig. 2C). All these transcripts appeared to be significantly upregulated in EGFP-positive cells compared with EGFP-negative ones, thus demonstrating the Stat3 responsiveness of the reporter line.

Taken together, these results highlight that *Tg(7xStat3:EGFP)* transgenic fish are a suitable biosensor for *in vivo* analyses of canonical Stat3 transcriptional activity.

### Stat3 pathway is active in proliferating cells during development and in adult intestine

**Fig. 3.**
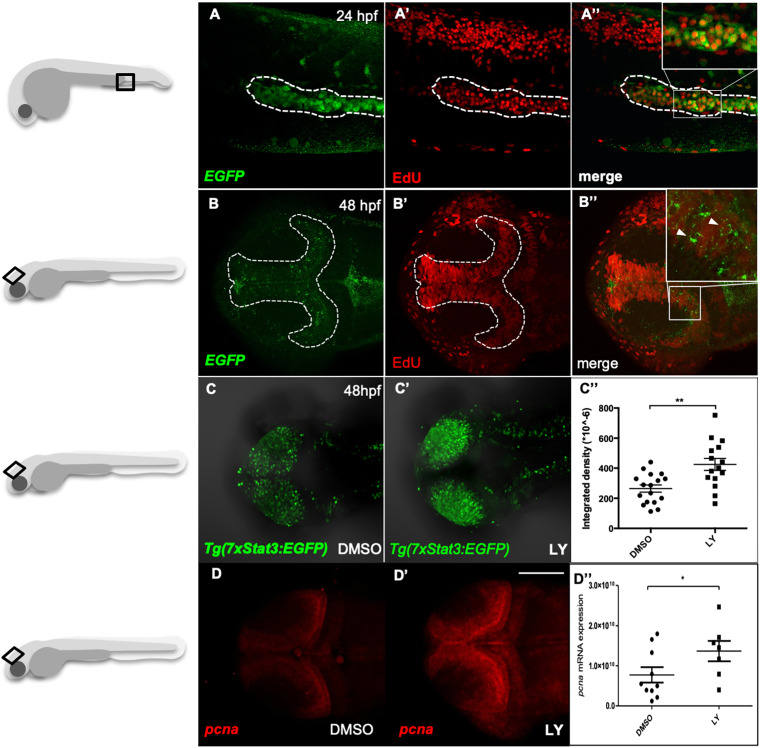
**Stat3 pathway is active in proliferating cells of zebrafish haematopoietic tissue and optic tectum.** (A-A″) Fluorescence co-localization (A″) using α-EGFP Ab (A) and EdU proliferation assay (A′) in the haematopoietic tissue (dashed line) of 22 hpf *Tg(7xStat3:EGFP)* reporter embryos. (B-B″) Fluorescence co-localization (B″) between fish using α-EGFP probe (B) and EdU proliferation assay (B′) in the optic tectum (TeO; dashed line) of 48 hpf embryos. (C,C′) *In vivo* fluorescence of *Tg(7xStat3:EGFP)* reporter activity in the TeO of embryos treated with LY364947 inhibitor between 24 and 48 hpf (C′) compared with DMSO-treated controls (C). (C″) Relative fluorescence intensity in the TeO of 48 hpf *Tg(7xStat3:EGFP)* embryos described in C (*n*=15, *P*=0.0014). (D,D′) Whole mount *in situ* hybridization detection of *pcna* mRNA in the TeO of 48 hpf embryos treated with LY364947 inhibitor between 24 and 48 hpf (D′) compared with DMSO-treated controls (D). (D″) *pcna* mRNA expression in the embryos described in D (*P*=0.038). All statistical analyses were performed by unpaired *t*-test; *P<0.05, **P<0.01. Graphs indicate mean±s.e.m. Scale bar: 100 μM.

The EGFP expression in *Tg(7xStat3:EGFP)* fish was mainly localized in those tissues that are known to be highly proliferating during zebrafish embryogenesis and larval development. To test whether EGFP-positive cells are involved in proliferative processes, we used EdU to label at 24 and 48 hpf the mitotic cells of *Tg(7xStat3:EGFP)* transgenic fish. The significant co-localization between EdU and EGFP in the haematopoietic niche (Fig. 3A-A″) and proliferating tectal zone (TPZ) of the PML (Fig. 3B-B″), indicates that the Stat3 pathway is indeed active in proliferating cells during zebrafish embryogenesis.

We have previously demonstrated that, during zebrafish organogenesis, TGFb is a powerful signal for cell cycle arrest (Casari et al., 2014). Hence, we decided to increase the proliferation rate of zebrafish larvae by inhibiting TGFb signalling and test the correlation between increased proliferation and Stat3 responsiveness. To this purpose, we treated *Tg(7xStat3:EGFP)* reporter embryos with an inhibitor of TGFb type I receptor (LY364947). As shown in Fig. 3C-C″, this stimulus to the cell cycle enhanced the levels of *Tg(7xStat3:EGFP)* fluorescence in the TeO. Consistently, *in situ* hybridization showed a significant increase in the expression of the proliferation marker *pcna* in LY364947-treated larvae compared with controls (Fig. 3D-D″). Taken together, these data link, once again, increased proliferation and Stat3 signalling.

**Fig. 4.**
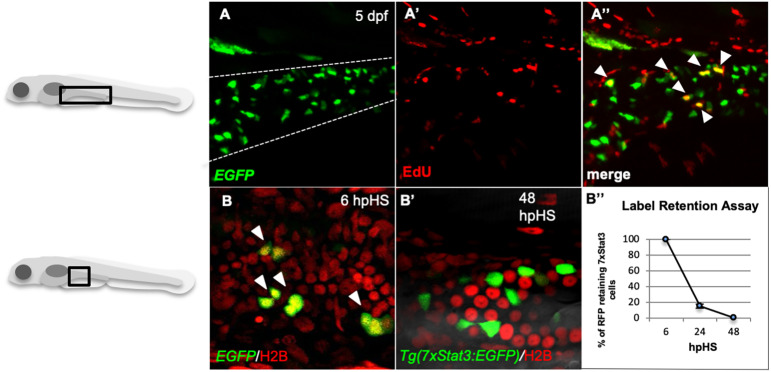
**Stat3 pathway is active in rapidly proliferating intestinal cells during zebrafish larval development.** (A-A″) Co-localization (A″) using α-EGFP Ab (A) and EdU proliferation assay (A′) in the developing intestine (dashed line) of 5 dpf *Tg(7xStat3:EGFP)* larvae. (B,B′) Label retention assay using double transgenic *Tg(7xStat3:EGFP)/Tg(HSP70:H2B:mRFP)* 5 dpf larvae. The ubiquitous RFP label, accumulated in 100% of intestinal cells at 6 hpHS, is completely lost in EGFP-expressing cells at 48 hpHS, where no EGFP/RFP co-localization is appreciable (B′). (B″) RFP loss by *Tg(7xStat3:EGFP)-*positive intestinal cells. Three independent biological replicates were performed.

The intestinal epithelium represents the most vigorously renewing tissue in mammals and zebrafish (Barker et al., 2007; Ng et al., 2005). Consistently, *Tg(7xStat3:EGFP)* reporter expression co-localized with EdU stain in the intestine of 5 dpf larvae (Fig. 4A-A″). To better understand the proliferation dynamics of Stat3-responsive cells, we also performed a label retention assay (Foudi et al., 2009). *Tg(7xStat3:EGFP)* fish were crossed with the histone-specific *Tg(HSP70:H2B-RFP)* line in order to follow the proliferation-dependent dilution of H2B-RFP nuclear signal in EGFP/Stat3-positive cells. Interestingly, after the first heat shock, which labels in red all larval cells, the rapid loss of nuclear RFP in all intestinal Stat3-positive cells was completed within 48 h post heat shock (hpHS). This result demonstrates that Stat3-expressing cells have an elevated rate of proliferation during intestinal development (Fig. 4B-B″).

Taken together, these data indicate that during the first days of zebrafish development Stat3 is mainly active in proliferating cells of the haematopoietic tissue, nervous system and intestine.

### Stat3 is active in stem-like intestinal cells and needed for normal gut development

**Fig. 5.**
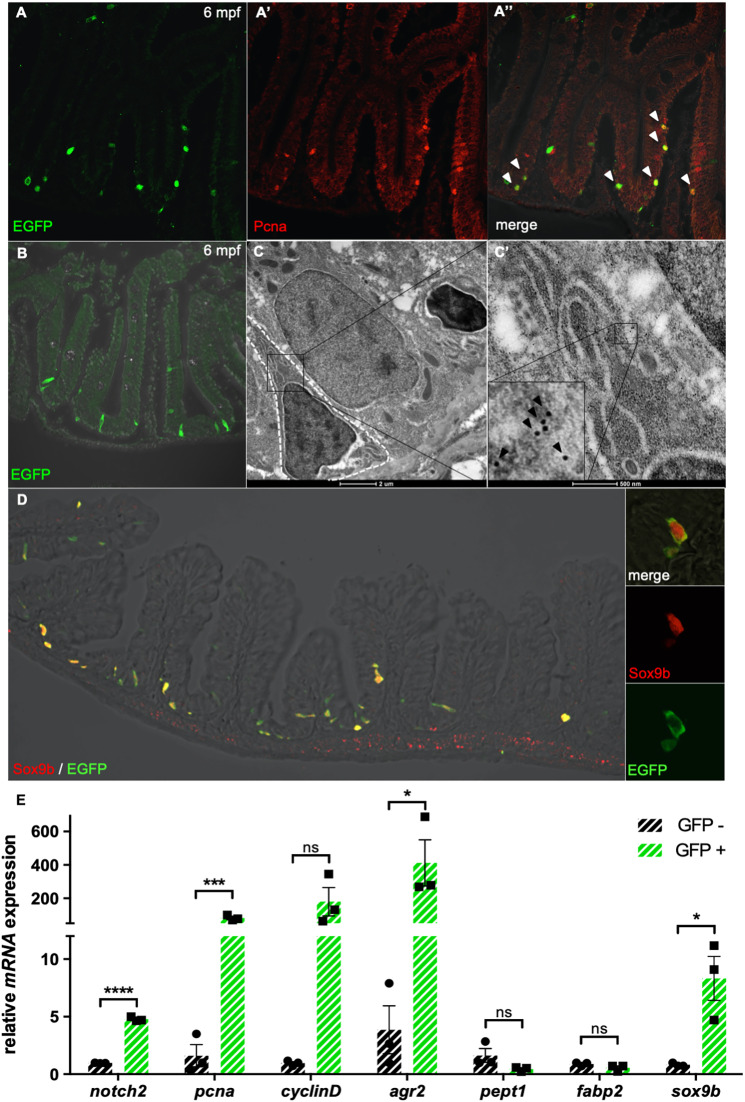
**Stat3 is active in intestinal FBC cells of adult zebrafish.** (A-A″) Co-localization (A″) using α-EGFP Ab (green) (A) and α-PCNA Ab (red) (A′) staining of adult *Tg(7xStat3:EGFP)* intestine. (B) Staining with α-EGFP Ab on a transversal section of adult *Tg(7xStat3:EGFP)* intestine, showing that all Stat3-positive cells are located at the base of the intervillus pocket. (C,C′) Immunogold staining with α-EGFP Ab on adult *Tg(7xStat3:EGFP)* intestinal sections observed by TEM. A gold-labelled cell is surrounded with a white striped line (C). High magnification of gold-labelled cytoplasm belonging to triangular-shaped fold base columnar (FBC) cell; inset is zoom of boxed area, showing gold dots (black arrowheads). (D) Staining with α-EGFP Ab (green) and α-Sox9b Ab (red) of adult *Tg(7xStat3:EGFP)* intestine*.* (E) qRT-PCR analysis of *notch2*, *pcna*, *cylcinD1*, *agr2*, *pept1*, *fabp2* and *sox9b* expression in EGFP-positive and EGFP-negative cells taken from adult intestines. Statistical analysis was performed by unpaired *t*-test; *P<0.05, ***P<0.001, ****P<0.0001; ns, not significant. Error bars indicate s.e.m.

When focusing our attention on the adult intestine, we observed that all EGFP-positive cells co-localized with the proliferation marker Pcna, thus confirming that Stat3-responsive intestinal cells retain their mitotic potential until adulthood (Fig. 5A-A″).

Notably, the self-renewal of the mammalian intestinal epithelium is fuelled by crypt base columnar (CBC) cells, a population of small, adult, undifferentiated, cycling stem cells active at the base of the mammalian crypt in the so-called ‘stem cell zone’ (Barker et al., 2007; Cheng and Leblond, 1974). Histological sections show that the anatomy and architecture of adult zebrafish intestinal tract closely resemble those of the mammalian small intestine (Ng et al., 2005); although lacking cryptae *sensu stricto*, they instead have functionally identical intervillus folds. Transgene expression analysis localized zebrafish Stat3 activity in a population of proliferating cells located at the base of the intestinal intervillus folds (Fig. 5B). To understand the nature of the Stat3-responsive cells, we performed a morphological analysis, based on immunogold labelling, in the intestine of *Tg(7xStat3:EGFP)* adult fish. Using an anti-EGFP antibody, we observed that Stat3 was active in some intestinal triangular-shaped cells with a global morphology resembling that of CBC, clearly characterized by a distinctive large dense nucleus that occupied most of the cell body (Fig. 5C,C′).

The transcription factor Sox9 is a known marker for mammalian intestinal stem cells expressed throughout the crypt; notably, its orthologue Sox9b was recently identified in medaka fish intestine as a molecular marker for stem cells at the base of intestinal folds (Formeister et al., 2009; Aghaallaei et al., 2016). To unequivocally identify zebrafish intestinal Stat3-expressing cells as CBC-like, we decided to stain the intestine of *Tg(7xStat3:EGFP)* with antibodies against the transcription factor Sox9b. More than 75% of the intestinal cells expressing EGFP under the control of Stat3-responsive elements also expressed Sox9b marker in their nuclei (Fig. 5D). Furthermore, the *sox9b* expression levels appeared tenfold higher in *Tg(7xStat3:EGFP)* intestine EGFP-positive cells than in EGFP-negative cells (Fig. 5E). These data suggest that the Stat3 pathway operates in a population of zebrafish CBC-like cells that we have named ‘fold base columnar’ (FBC) cells.

To better understand the characteristics of *Tg(7xStat3:EGFP)* intestinal fluorescent cells, we performed qRT-PCR on sorted cells samples and analysed the expression levels of some well-known intestinal genes: *fabp2*, *pept1* and *agr2* (Fig. 5E). The protein Fabp2 is member of a family of intestinal lipid-binding proteins (Esteves et al., 2016) and is present in intestinal absorptive cells (Gajda and Storch, 2015). No significant differences in *pept1* and *fabp2* transcript levels were detected between EGFP-positive and EGFP-negative cells; however, *agr2*, reported as a stem cell maker regulated by the Wnt/β-catenin canonical signalling pathway (Dahal Lamichane et al., 2019), appears upregulated in EGFP-positive cells with respect to EGFP-negative ones. These data indicate higher levels of Wnt/β-catenin activity and stemness traits in EGFP fluorescent cells. We also decided to measure the expression levels of the proliferation markers *pcna* and *cyclinD1* and found that they were increased in EGFP-positive cells, revealing that they were actively proliferating. Moreover, Notch has been recently identified as a key regulator of stemness in the intestine (Chen et al., 2017); we observed an increase in *notch2* transcripts levels in *Tg(7xStat3:EGFP)* intestinal cells compared with *Tg(7xStat3:EGFP)*-negative cells, confirming that EGFP-positive cells are intestinal stem cells.

To exclude the secretory nature of the fluorescent cells in the gut, we crossed the *Tg(7xStat3:EGFP)* reporter line with the *Tg(nkx2.2a:mEGFP)* transgenic zebrafish line, in which enteroendocrine cells of the intestine are labelled with membrane EGFP (Ng et al., 2005). Interestingly, Stat3-dependent fluorescent cells and *Tg(nkx2.2a:mEGFP)*-positive cells are different populations, as clearly revealed by both cell counting and morphological analysis under fluorescent confocal microscopy (Fig. S6A,B). These data show that *Tg(7xStat3:EGFP)* intestinal fluorescent cells are not enteroendocrine cells, and the morphology of EGFP-positive cells revealed by fluorescent and electron microscopy (Fig. 5A,C) also excludes the possibility that they might be goblet cells.

**Fig. 6.**
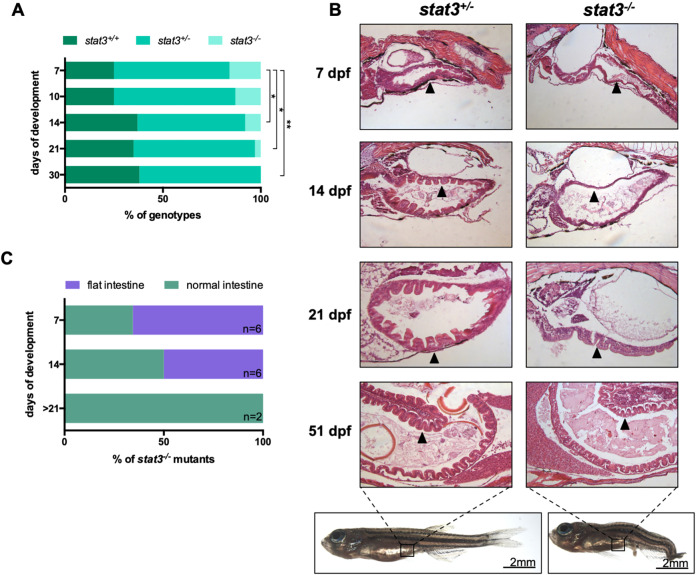
**Stat3 is required for intestinal homeostasis and larvae survival during development.** (A) Percentage of the different genotypes found at different time points in *stat3^+/−^* incross; *stat3*^−/−^ mutants were never found after 25 dpf, with the exception of a single animal that died at 51 dpf (shown in B). Statistical analysis was performed by chi-square test to compare proportions. (B) Haematoxylin-eosin staining of longitudinal sections at different larval stages (indicated on the left); arrowhead indicates the intestinal epithelium. Compared with normal fish, mutants display flattening of the intestinal epithelium. (C) Percentage of individuals at different stages presenting either normal intestine or degenerated intestine lacking intestinal folds. *P<0.05; **P<0.01.

To assess the requirement for Stat3 in the development and maintenance of the intestinal epithelium, we analysed gut formation in *stat3*^−/−^ mutants. The *stat3^ia23^* allele was genetic lethal, with the humane endpoint reached between 7 and 21 dpf (Fig. 6A). However, out of hundreds of fish screened for homozygosity at later stages, we observed only two surviving *stat3^−/−^* mutants, which survived until 21 and 52 dpf, respectively (Fig. 6A,B). For the characterization of genotyped fish, we used a classical histochemical approach focusing on the anatomy of the intestinal epithelium. In general, the most prominent phenotype of mutant larvae was the complete lack of intestinal folds; in particular, quantitative analysis at 7 dpf showed an incomplete penetrance of this phenotype (about 67% of *stat3^−/−^* larvae) (Fig. 6A). However, at 14 dpf, only 50% of surviving mutant larvae lacked intestinal folds (Fig. 6B,C). This finding, combined with the fact that the proportion of *stat3*^−/−^ mutants decreased with time (Fig. 6A), suggests that incomplete gut development might be the leading cause of *stat3^−/−^* mutant mortality. Indeed, the two mentioned survivors (21 and 52 dpf) displayed a normal intestinal mucosa (Fig. 6B).

### Canonical Wnt signalling drives proliferation of Stat3-responsive intestinal cells

**Fig. 7.**
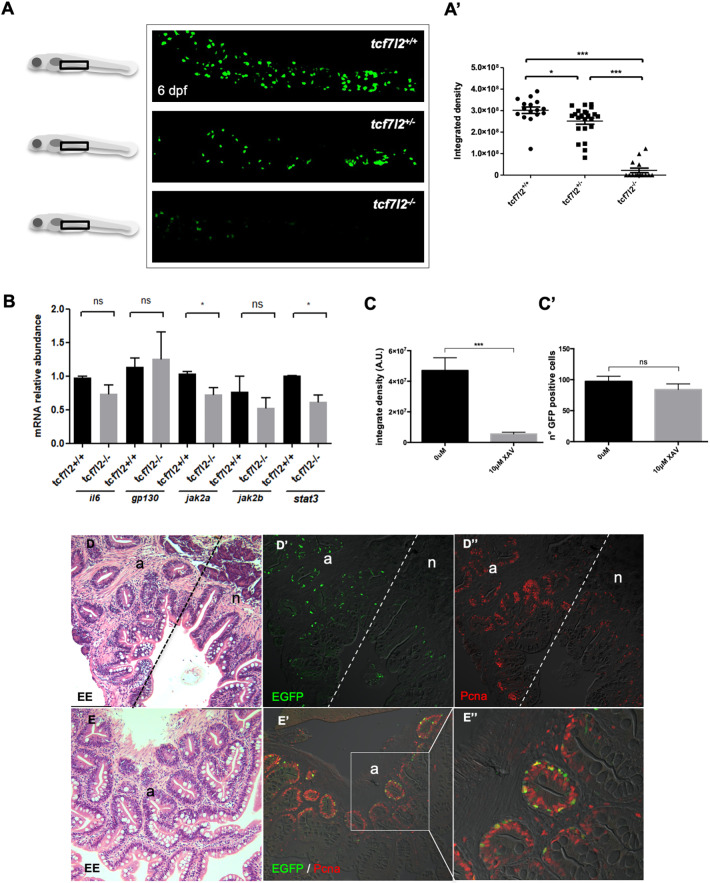
**Tcf7l2 (Tcf4) is required for development of Stat3-responsive cells of zebrafish larvae intestine and the Stat3 pathway is activated ectopically in intestinal adenomas of *apc^hu745^* mutants.** (A,A′) *In vivo* EGFP fluorescence in the intestine of 6 dpf *Tg(7xStat3:EGFP)*/*tcf7l2^hu892^*^/*hu892*^, *Tg(7xStat3:EGFP)*/*tcf7l2*^+/*hu892*^ and *Tg(7xStat3:EGFP)*/*tcf7l2*^+/+^ siblings (A) and measurement of integrated density (A′) (*n*=16). (B) qPCR analysis of *il6*, *gp130*, *jak2a*, *jak2b* and *stat3* mRNA expression from *tcf7l2^+/+^* and *tcf7l2^hu892/hu892^* sibling larvae. (C,C′) Effect of XAV treatment on *Tg(7xStat3:EGFP)* embryos from 48 to 78 hpf: measurement of the integrated density of the fluorescence (C) and measurement of the number of GFP^+^ cells (C′). (D,E) Haematoxylin-eosin staining on paraffin embedded transversal section of zebrafish *apc^hu745^* intestine at 12 months post fertilization, displaying both normal tissue (n) and hyperplastic adenomas (a). (D′,D″) Staining of a sequential intestinal section of D using α-EGFP (green) (D′) and α-PCNA (red) (D″) Abs. (E′,E″) Double staining using α-EGFP Ab (green) and α-PCNA Ab (red) of a sequential intestinal section of E. All statistical analyses were performed by unpaired *t*-test. *P<0.05, ***P<0.001; ns, not significant. Error bars indicate s.e.m.

It is known that T cell factor 4 (Tcf7l2, also known as Tcf4) is a Wnt/β-catenin transducer important in preserving proliferative self-renewal in the zebrafish intestine throughout life (Muncan et al., 2006), like its murine orthologue (Korinek et al., 1998). In addition, Sox9 in the mammalian intestinal crypts is a direct target of β-catenin/Tcf7l2 (Blache et al., 2004). Furthermore, *Tcf4^hyg/hyg^* knockout mice show the total abrogation of small intestine proliferative cells of the intestinal crypts (van Es et al., 2012). Hence, we enquired whether the Stat3-active cells of the zebrafish FBC also depend on β-catenin/Tcf7l2 signalling. To this purpose, we crossed *Tg(7xStat3:EGFP)* individuals with *tcf7l2^hu892^* carriers to generate *Tg(7xStat3:EGFP)* transgenics in a *tcf7l2* mutant background. As shown in Fig. 7, the EGFP level in the Stat3 reporter was almost abolished in the mutants, showing once again that the Stat3-responsive cells in the gut of *Tg(7xStat3:EGFP)* are indeed stem cells of the zebrafish intestine. Moreover, we treated *Tg(7xStat3:EGFP)* embryos with XAV, a Wnt/β-catenin inhibitor commonly used in zebrafish (Moro et al., 2012). Interestingly, in embryos treated from 48 hpf (before intestinal organogenesis) to 78 hpf (when the primordial intestinal tube is formed) we found a strong decrease in activation of the Stat3 reporter line (Fig. 7C) and, on the other hand, a non-significant decrease in the number of EGFP-positive cells (Fig. 7C′). Moreover, we performed qRT-PCR analysis on EGFP-positive cells sorted from adult intestines for detection and quantification of the known intestinal Wnt receptors *fzd5* and *fzd8a* (Nikaido et al., 2013). Significantly higher level of expression of these two transcripts, compared with EGFP-negative cells, confirmed the direct responsiveness of EGFP-positive cells to Wnt ligands (Fig. S5A). These results suggest an important role for Wnt/β-catenin signalling in the intestine, being needed for the maintenance of Stat3 activity in FBC cells but not essential for formation of these cells.

To investigate further the correlation between β-catenin/Tcf7l2 signalling and Stat3, we performed qPCR on 6 dpf *tcf7l2^+/+^* and *tcf7l2^hu892/hu892^* larvae and analysed the gene expression levels of members of the Stat3 pathway (Fig. 7B). The *tcf7l2^hu892/hu892^* mutants showed an overall downregulation of the pathway: in particular, *jak2b* and *stat3* mRNA levels appeared to be significantly downregulated in mutants compared with wild-type siblings, suggesting a role for β-catenin/Tcf7l2 signalling as a key regulator of the Stat3 pathway.

To study *in vivo* the correlation between Wnt signalling and Stat3-dependent intestinal proliferation in more depth, we took advantage of the zebrafish *apc^hu745^* mutant allele that, being unable to form the β-catenin destruction complex, displays ubiquitous canonical Wnt signalling pathway activation. Although *apc^hu745/hu745^* mutants die before 4 dpf, *apc^hu745/+^* adults, similarly to the mammalian model, develop spontaneous highly proliferating adenomatous polyps in the intestine, by loss of heterozygosis (Haramis et al., 2006). Notably, when intestinal polyps developed in *apc^hu745^*/*Tg(7xStat3:EGFP)* mutants*,* the fish gut displayed a significant increase in EGFP fluorescence compared with normal tissue (Fig. S5B,C). Anti-EGFP immunofluorescence analysis on *apc^hu745^* intestinal sections showed an enrichment in the number of Stat3-responsive cells within the adenomatous polyps (Fig. 7D,D′, left side), whereas Stat3 activity was present only in a few isolated cells at the base of the intestinal folds in normal tissue (Fig. 7D,D′, right side). Consistently, Pcna proliferation marker was also enriched in adenomatous regions with high Stat3 activity (Fig. 7D,D″). Notably, co-localization between Stat3 and Pcna revealed that all Stat3-positive cells had an active cell cycle, whereas Stat3 was active only in a portion of Pcna-expressing cells (Fig. 7E′,E″). In conclusion, Stat3 activity strictly correlates with proliferation in both normal and adenomatous zebrafish intestines.

Finally, to test whether Stat3 and canonical Wnt signalling are entangled in an autocrine sustaining loop, a feature commonly used by genetic cascades to maintain the stem niche, we treated a Wnt/β-catenin reporter line (Moro et al., 2012) with the Jak2 inhibitor AG-490. As shown in Fig. S5D, we could not detect a significant effect of Jak2 inhibition on the global levels of canonical Wnt signalling, thus suggesting a one-way signal transduction cascade from Wnt to Stat3.

## DISCUSSION

In this work, a Stat3 reporter and a Stat3 CRISPR/Cas9 zebrafish mutant were generated, validated and analysed to provide a broad view of Stat3 activities and essential functions during early development, tissue homeostasis and pathogenesis. We designed the Stat3-specific reporter cassette based on previous studies performed in mammalian cells, in which a 7× tandem repeat containing the CRP-APRE sequence (TTCCCGAA) from the human CRP promoter was shown to respond to IL-6-mediated Stat3 transcription (Zhang et al., 1996). Consistently, the fluorescence of the *Tg(7xStat3:EGFP)* reporter was inhibited by Jak2 tyrosine kinase inhibitors and, instead, was activated by injection of dominant active forms of either *stat3* or IL-6 (data not shown). Unexpectedly, although significantly reduced, some fluorescence of *Tg(7xStat3:EGFP)* was also maintained in a Stat3 null background. This might be due to the fact that in the absence of Stat3, and the resultant negative feedback mechanisms activated through Socs1 protein (Liau et al., 2018), Jak2 can phosphorylate other Stat proteins. As a consequence, activated Stats, other than Stat3, can bind Stat3-responsive elements with lower transcriptional efficiency and maintain some EGFP expression as well as activate essential Stat3 target genes. Moreover, *stat1a* transcript levels appeared to be higher in *stat3^−/−^* fish than in *stat3^+/+^* siblings (Fig. S4D), suggesting an involvement of Stat1a in this possible compensation mechanism. Consistent with this, when the Jak2-mediated activation of Stats was inhibited with AG-490, the EGFP expression of *Tg(7xStat3:EGFP)* was completely abolished*.*

The Stat3-dependent fluorescence was already detectable in the eggs of *Tg(7xStat3:EGFP)* fish. This finding is in agreement with the maternal requirement for Stat3 in embryonic cell proliferation and axis extension at gastrulation (Liu et al., 2017). Notably, the early Stat3 transcriptional activity in zebrafish is also consistent with the embryonic lethality of Stat3 null mice (Takeda et al., 1997) and the requirement of LIF/Stat3 for the maintenance of naïve pluripotency in mouse embryonic stem cells (Carbognin et al., 2016). Moreover, embryos of the stable *Tg(7xStat3:EGFP)* line display a fluorescent pattern that significantly overlaps the zebrafish *stat3* mRNA expression profile previously described by Thisse et al. (2004). In particular, the *Tg(7xStat3:EGFP)* reporter is active in telencephalon, retina, cranial ganglia, lateral line system, optic tectum, haematopoietic niche and intestine, all tissues in which Stat3 has been reported to play key roles (Levy and Lee, 2002; Pickert et al., 2009). During organogenesis, the *Tg(7xStat3:EGFP)* reporter activity was particularly elevated in regions with a high proliferative index, such as the larval intestine, the primary haematopoietic tissue and the posterior region of the optic tectum. In all these regions, Stat3-positive cells were themselves actively proliferating, as demonstrated by label retention assay, co-localization with Pcna and EdU labelling. The fact that these actively proliferating tissues expressed high levels of Stat3 is in agreement with the well-known oncogenic activity of Stat3 in glioblastoma, colorectal cancer and leukemia (Sherry et al., 2009; Corvinus et al., 2005; Benekli et al., 2002).

Stat3 responsiveness in the adult zebrafish intestine was located in a restricted population of actively proliferating cells identified as FBC cells; remarkably, FBC cells expressed the stem cell specific marker Sox9b and displayed a stem cell-like morphology. These findings are strongly consistent with Stat3 activity being absolutely required in mice for small-intestine crypt stem cell survival at both the +4 to +6 label-retaining and crypt base columnar cell locations (Matthews et al., 2011). On the other hand, it is known that mice carrying conditional ablation of Stat3 in differentiated intestinal epithelial cells develop normally (Pickert et al., 2009). Hence, mouse and zebrafish findings are totally compatible with the hypothesis that Stat3 is required for survival and expansion of intestinal progenitors. Notably, both the AG-490 treatment and *stat3^ia23^* mutation led to a severe impairment of intestine development that might well be the cause of loss of all *stat3^ia23/ia23^* fish before the third week of age. Thus, slightly different from the observations of Liu et al. (2017) in their zebrafish *stat3* knockout mutant, our results drive the conclusion that Stat3 is needed for early larval development, being functionally conserved and needed for gut development and maintenance. The highly proliferating Sox9b-positive and Stat3-responsive cells of zebrafish intestine require Tcf/β-catenin for their development and are extremely expanded in number in intestinal adenomas of *apc* mutant fish, hence indicating that Stat3 responsiveness and its consequence in the gut are under control of the Tcf/β-catenin signalling pathway. Moreover, the fact that the Stat3-responsive cells of zebrafish intestinal adenomas represent only a fraction of the proliferating population suggests that they might be intestinal cancer stem cells, thus denoting Stat3 as a stemness marker in normal and neoplastic intestine, and Jak2 kinase as a suitable therapeutic target in CRC.

## MATERIALS AND METHODS

### Animal husbandry and lines

Animals were staged and fed as described by Kimmel et al. (1995) and maintained in a large-scale aquaria system.

Embryos were obtained by natural mating, raised at 28°C in Petri dishes containing fish water (50×; 25 g Instant Ocean, 39.25 g CaSO_4_ and 5 g NaHCO_3_ for 1 l) and kept in a 12:12 light-dark cycle. All experimental procedures complied with European Legislation for the Protection of Animals used for Scientific Purposes (Directive 2010/63/EU).

*apc^hu745^* (Haramis et al., 2006) mutant carriers were genotyped by PCR amplification and sequencing. The *Tg(HSP70:H2B:mRFP)* transgenic line was a kind gift of the Meyer Lab (Institute of Molecular Biology, Leopold-Franzens-University Innsbruck). The *Tg(gata1:dsRed)^sd2^* transgenics, *Tg(nkx2.2a:mEGFP)* transgenic lines and *tcf4^hu892^* (*tcf4^exI^*^/*exI*^) mutants are described by Traver et al. (2003), Ng et al. (2005) and Muncan et al. (2006), respectively. The *Tg(7xTCF-Xla.Siam:nlsmCherry)^ia5^* reporter zebrafish line is described by Moro et al., 2012. All animal experiments were performed under permission of the ethical committee of the University of Padova and the Italian Ministero della Salute (23/2015-PR).

### Generation of *Tg(7xStat3-Hsv.Ul23:EGFP)* reporter and *stat3^ia23^* mutant

The Stat3-responsive promoter sequence, containing the seven tandem repeats from the promoter of human CRP (nucleotides −123 to −85), was obtained in the form of the pLucTKS3 plasmid, a kind gift of Prof. Jiayuh Lin (The Ohio State University Comprehensive Cancer Center, Columbus, OH). The promoter fragment, containing the SBEs and the TK minimal promoter, was isolated from the pLucTKS3 plasmid using *Hin*dIII and *Sac*II restriction enzymes and subcloned into the Gateway 5′ entry vector pME-MCS (Invitrogen). The resulting p5E-TKS3 entry vector was recombined, together with the EGFP-carrying middle entry vector and the p3E-polyA entry clone, into the Tol2 destination vector pDestTol2pA2 (Invitrogen) as already described (Kwan et al., 2007). We co-injected 30 pg of the recombined Tol2 destination vectors with 25 pg of *in vitro* synthesized Tol2 transposase mRNA (Kawakami et al., 2004) into wild-type zebrafish embryos at the one-cell stage. Microinjected embryos were selected at 24 hpf for their elevated mosaic transgenic expression using an epifluorescent microscope, raised to adulthood and outcrossed to wild-type fish. The F_1_ founders were finally selected for fluorescence level and Mendelian segregation of their transgene in order to establish single allele transgenic lines *Tg(7xStat3-Hsv.Ul23:EGFP)*.

The *stat3^ia23^* zebrafish mutants were generated by CRISPR/Cas9-mediated genome editing. A single guide RNA (sgRNA) was designed using the CHOPCHOP software (https://chopchop.rc.fas.harvard.edu) to specifically target an optimal CRISPR sequence on exon 14 of the *stat3* gene (NM 131479). The *stat3*-targeting sgRNA (with the specific targeting sequence GGTCGATCTTAAGTCCTTGG-ngg), was generated according to Gagnon et al. (2014) and transcribed *in vitro* using the MEGAshortscript T7 kit (Life Technologies, AM1354).

One-cell stage embryos were injected with 2 nl of a solution containing 280 ng/µl Cas9 protein (M0646, Bio Labs) and 68 ng/µl sgRNA; Phenol Red was used as injection marker. F_0_ injected embryos were raised to adulthood and screened, by genotyping the F_1_, for germline transmission of the mutation. Heterozygous mutants harbouring the mutation were then outcrossed four times and then incrossed to obtain homozygous mutants (F_5_ generation).

*stat3^ia23^* heterozygous and homozygous mutants were identified by loading in agarose gel the product of the PCR performed using the following primers: *stat3*-forward 5′-GGCCTCTCTGATAGTGACCG-3′ and *stat3*-reverse 5′-AGTTGTGCTTAGACGCGATC-3′.

### Organ dissection

To analyse the reporter signal in the adult, *Tg(7xStat3-Hsv.Ul23:EGFP)* fish were euthanized and dissected under the epifluorescence microscope. The intestine and other organs belonging to the gastro-urinary tract were imaged immediately after, either *in situ* or isolated, with conventional fluorescence or confocal microscopy. Isolated intestine from *Tg(7xStat3-Hsv.Ul23:EGFP)/apc^+/−^* and *Tg(7xStat3-Hsv.Ul23:EGFP)* zebrafish at 12 months post fertilization (mpf) were dissected in PBS solution and then fixed in 4% PFA in PBS for paraffin embedding, cryosectioning or further immunolabelling.

### Immunofluorescence, immunogold labelling and TEM analysis

Immunofluorescence staining on sections of 6 mpf *Tg(7xStat3-Hsv.Ul23:EGFP)*/*apc*^+/−^ intestines was performed as previously described by Schiavone et al. (2014) using chicken anti-EGFP antibody (1:250; A10262, Life Technologies), mouse anti-PCNA (1:100; M0879, Dako), anti-chicken-AlexaFluor488 (1:500; A-11039, Life Technologies) and anti-mouse-TRITC (1:500; R 0270, Dako). Cryosectioning and immunofluorescence analysis of 6 mpf *Tg(7xStat3-Hsv.Ul23:EGFP)* intestines were performed as described by Zhang et al. (2017) using goat anti-Sox9b (zebrafish) (1:100; ER14-1692, RayBiotech).

Whole mount immunofluorescence staining was carried out on zebrafish larvae injected with CMV-mStat3C plasmid. At 24 hpf, larvae were fixed with 4% PFA for 2 h at room temperature and stored at −20°C in methanol. After rehydration, larvae were washed with 150 mM Tris-HCl (pH 9) for 5 min. Then the samples were incubated at 70°C for 15 min. After two 5 min washes in PBS containing 1% Triton X-100, larvae were permeabilized with acetone for 20 min at −20°C. Acetone was removed with two washes in deionized water and two washes in PBS containing 1% Triton X-100. Epitopes were blocked for 2 h with PBS containing 1% Triton X-100, 1% BSA and 2% goat serum. Samples were then incubated with primary antibody anti-Stat3 mouse monoclonal (1:75; Cell Signaling, 9139) for 72 h. After a brief wash in PBS with 1% Triton X-100, samples were washed twice with PBS containing 1% Triton X-100 and 5% goat serum for 1 h. After overnight incubation of the samples with secondary antibody (anti-mouse AlexaFluor488, Life Technologies), larvae were washed twice with PBS containing 1% Triton X-100 and 5% goat serum for 1 h and three times with PBS containing 0.5% Triton X-100 for 10 min.

Immunogold labelling was performed on adult *Tg(7xStat3-Hsv.Ul23:EGFP)/apc^+/−^* zebrafish intestine using chicken anti-EGFP (1:25; A10262, Life Technologies) primary antibody and anti-chicken-(10 nm) Au secondary antibody (25429, EMS). TEM acquisitions were performed using a FEI Tecnai G2 microscope and a TVIPS F114 bottom-mounted camera.

### Drug treatments

The following chemical compounds were used: AG-490 (T3434, Sigma), LLL12 (573131, Calbiochem), LY-364947 (L6293, Sigma) and XAV939 (X3004, Sigma). All drugs were dissolved in DMSO, stored in small aliquots and kept in the dark at −20°C. *Tg(7xStat3-Hsv.Ul23:EGFP)* embryos were dechorionated and exposed to drugs diluted in fish water. Pigmentation was inhibited with 2 mM 1-phenyl-2-thiourea (PTU). Drug treatments were as follows: AG-490 (100 µM) from 24 to 48 hpf, AG-490 (60 µM) at 3-6 dpf, AG-490 (80 μM) from 8 to 72 hpf, LLL12 solution (0.025 µM) from 24-48 hpf, LY-364947 (60 µM) from 24 to 48 hpf and XAV939 (10 μM) from 48-78 hpf.

### Proliferation assays

EdU proliferation assay was performed using the Click-iT EdU Alexa Fluor 594 Imaging Kit (Thermo Fisher Scientific) following the manufacturer's instructions. After initial exposure to EdU reagent, *Tg(7xStat3-Hsv.Ul23:EGFP)* 48 hpf embryos and 4 dpf larvae were kept in fish water for 1 h 30 min and 24 h, respectively. Proliferating cells were later stained with Alexa Fluor 594 red fluorophore and fixed in 4% PFA in PBS. Label retention assays were performed using the *Tg(HSP70:H2B:mRFP)* transgenic line. Offspring were heat-shocked at 4 dpf by replacing the fish water with water preheated to 42°C and afterwards incubating the larvae in an air incubator at 37°C for 1 h. The larvae were then analysed under the epifluorescent microscope for nuclear RFP and Stat3:EGFP fluorescence. Samples were mounted on a depression slide and monitored at 6, 24 and 48 hpHS to observe the dilution of nuclear staining using a Nikon C2 confocal microscope.

### *In situ* hybridization

Whole mount RNA *in situ* hybridization on zebrafish embryos was performed as described by Thisse and Thisse (2014), followed by staining with NBT/BCIP solution (Sigma, 11681451001). It is worth mentioning that treated and control embryos were hybridized together, as the control embryos had the tip of the tail cut for their *post hoc* recognition. The *EGFP* probe was synthesized from the pME-*EGFP* plasmid supplied by the Tol2 kit (Invitrogen). The *pcna* probe was obtained as described by Baumgart et al. (2014). The intensity of *in situ* hybridization signals was quantified after image segmentation based on colour hue, saturation and intensity using ImageJ software.

### mRNA isolation and qRT-PCR

For expression analysis, RNA was extracted from pools of 15 larvae at 7 dpf with TRIzol reagent (Thermo Fisher Scientific, 15596018). Total mRNA was treated with RQ1 RNase-Free DNase (Promega, M6101) and then used for cDNA synthesis with random primers (Promega, C1181) and M-MLV Reverse Transcriptase RNase H (Solis BioDyne, 06-21-010000) according to the manufacturer's protocol. qPCRs were performed in triplicate with the CybrGreen method using a Rotor-gene Q (Qiagen) and the 5× HOT FIREPol EvaGreen qPCR Mix Plus (Solis BioDyne, 08-36-00001); zebrafish *gapdh* was used as internal standard in each sample. The cycling parameters were 95°C for 14 min, followed by 45 cycles of 95°C for 15 s, 60°C for 35 s and 72°C for 25 s. Threshold cycles (Ct) and melting curves were generated automatically by Rotor-Gene Q series software. Ct values were divided for *gapdh* Ct values in order to obtain dCt values. As reported by Livak and Schmittgen (2001), one dCt was identified as a reference dCt and was subtracted from the dCt of other samples; with this procedure we obtained ddCt values. Results (*R*) were obtained with the following formula: *R*=2^ddCt^. Sequences of specific primers used in this work for qRT-PCR and RT-PCR are listed in Table S1. Primers were designed using the software Primer 3 (http://bioinfo.ut.ee/primer3-0.4.0/input.htm).

### Imaging

For *in vivo* imaging, transgenic embryos and larvae were anaesthetized with 0.04% tricaine, embedded in 0.8% low-melting agarose and mounted on a depression slide. The Nikon C2 confocal system was used to acquire fluorescent images from embryos and larvae and the immunofluorescence on slides. For *Tg(gata1:dsRed)sd2*/*Tg(7xStat3-Hsv.Ul23:EGFP)*, fish images were acquired using a Leica SP5 confocal microscope. Samples from *in situ* hybridization were mounted in 87% glycerol, then observed with a Leica M165 FC microscope equipped with a Nikon DS-Fi2 digital camera. Histological sections after haematoxylin-eosin staining were photographed on a Leica DMR using a Nikon DS-Fi2 digital camera. All images were analysed with Fiji (ImageJ) software and the integrated density of fluorescence was calculated, setting a standard threshold on non-fluorescent embryos.

VIBE-Z software (Ronneberger et al, 2012) was used to visualize single planes of the brain of 72 hpf *Tg(7xStat3-Hsv.Ul23:EGFP)* transgenic line.

### Fluorescence-activated cell sorting

*Tg(7xStat3-Hsv.Ul23:EGFP)* intestines were cut longitudinally in PBS and then were scraped in order to separate intestinal luminal cells from intestinal muscular tissue. Intestinal cells were treated with collagenase (2.2 mg/ml), trypsin (0.25 mg/ml) and EDTA (1 mM) in sterile PBS for 5 min and mechanically dissociated every minute. Dissociation was blocked by adding CaCl_2_ (1 mM) and 10% FBS. The cell suspension was filtered with a 100 μm cell strainer. After centrifugation at 800× ***g*** for 5 min, samples were resuspended in 200 μl of PBS containing 0.25 mg/ml trypsin and 1 mM EDTA and filtered through a 40 μm cell strainer. Then, cells were centrifuged at 800× ***g*** for 5 min and resuspended in 100 μl of sterile PBS containing 1% FBS, streptomycin 1× and 1 mM EDTA. For sorting we used a FACS Aria IIIu sorter (BD Biosciences, San José, USA) with the following settings for EGFP: argon-ion Innova Laser (Coherent, USA) (488 nm, 100 mW), 100 μM nozzle, sorting speed 500 events/s in 0-32-0 sort precision mode. We performed data acquisition and analysis with the BD FACSDiva software (BD Biosciences, San José, CA, USA).

### Statistical analysis

Statistical analysis was performed using Graph Pad Prism software V6.0. Data are presented as the mean±s.e.m. Comparison between different groups of samples was performed using Student's *t*-test with a confidence interval of 95%. The *P*-values are indicated with asterisks: *P<0.05, **P<0.01, ***P<0.001, ****P<0.0001.

## Supplementary Material

Supplementary information
