# Supplementary material for: The stem-like Stat3-responsive cells of zebrafish intestine are Wnt/β-catenin dependent
Source: Development. 2020 Jun 19;147(12):dev188987. doi: 10.1242/dev.188987 (PMC7328161; doi:10.1242/dev.188987)
Supplement: Supplementary information [file develop-147-188987-s1.pdf]

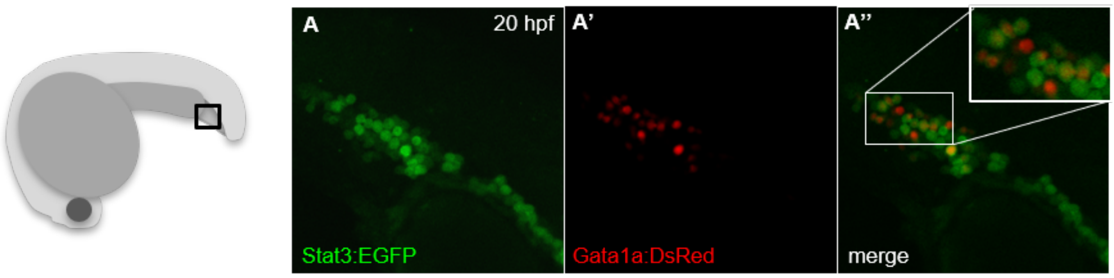


**Fig. S1: Stat3 reporter is expressed in erythroid progenitor cells.** A-A'': Confocal lateral view of haematopoietic tissue of double transgenic embryos obtained crossing the *Tg(7xStat3:EGFP)* (A) with *Tg(gata1a:DsRed)* (A'').

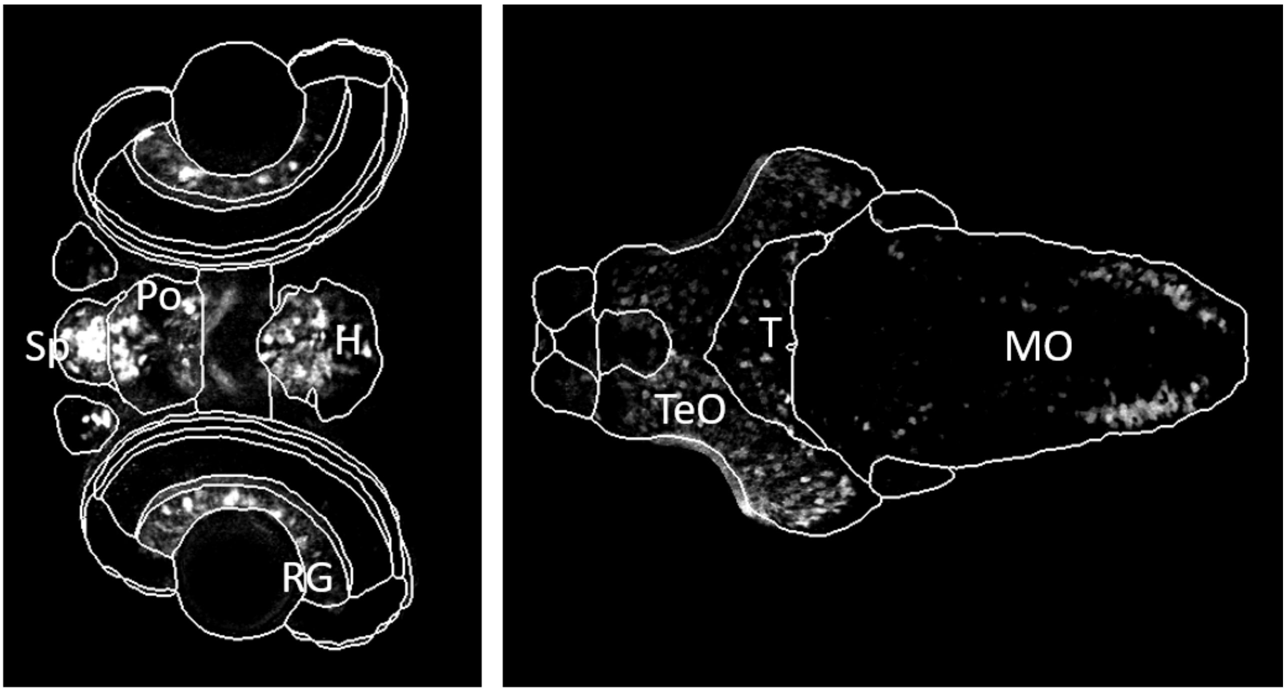


77 Tectum opticum (TeO)
97 Medulla oblongata (MO)
81 tegmentum (T)
7 subpallium (Sp)
45 preoptic region (Po)
48 hypothalamus (H)
59 retinal layer (RG)

**Fig. S2:** ***Tg(7xStat3:EGFP) reporter expression in the brain of 72-hpf larvae.*** A-B: Single planes of *Tg(7xStat3:EGFP)* brain at 72 hpf. Images have been obtained with VIBEZ-Z software. H= hypothalamus, Sp= subpallium, Po= preoptic region, RG= retinal layer, TeO= tectum opticum, MO=medulla oblongata, T= tegumentum.

Development • Supplementary information

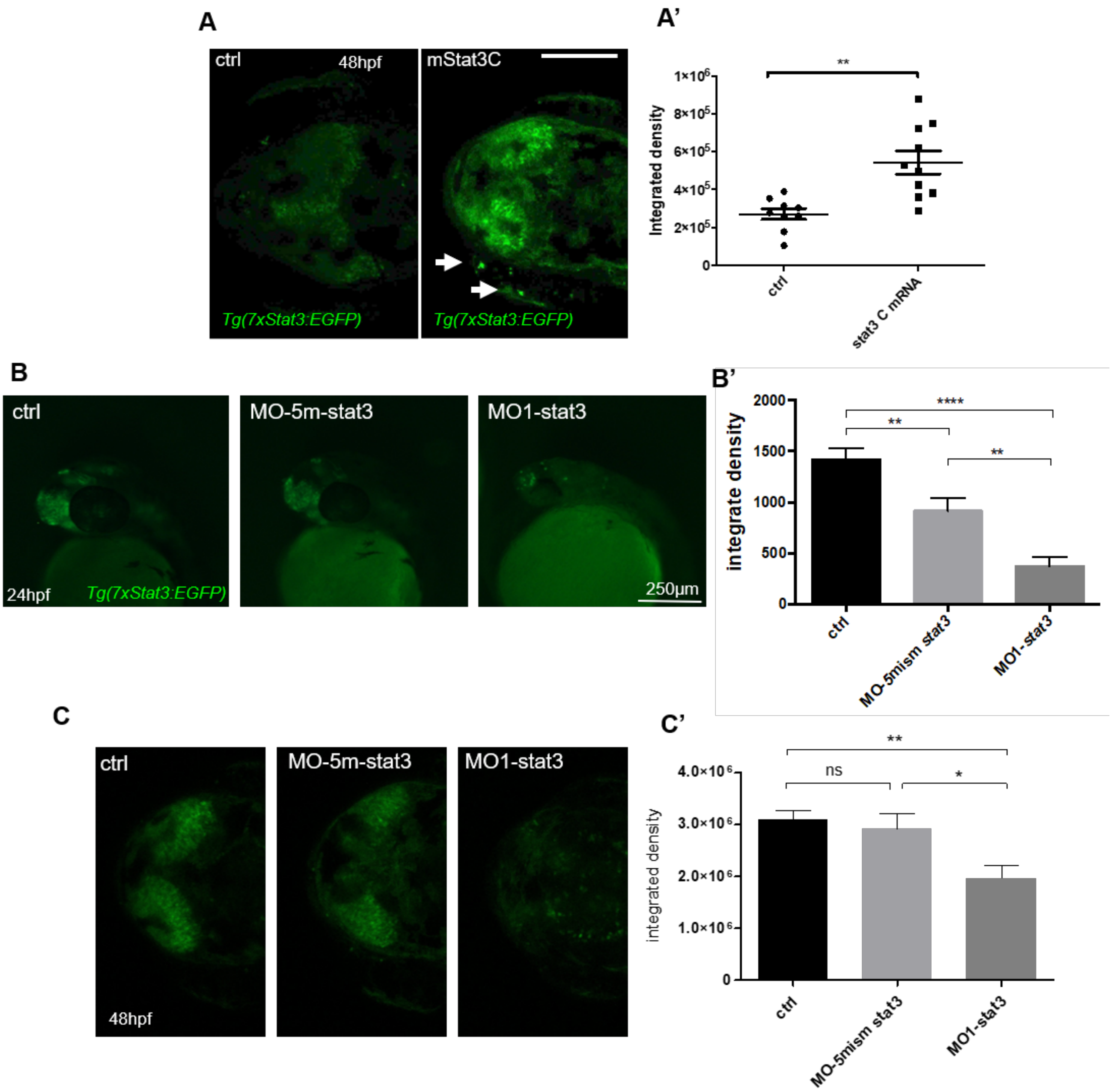


**Fig. S3: *Tg(7xStat3:EGFP)* reporter line respond to silencing and overexpression of *stat3*.** A: Dorsal view live image of the head of *Tg(7xStat3:EGFP*) controls (left) or m*Stat3C* mRNA injected embryos (right) at 48hpf. Arrowheads highlight ectopic fluorescent signal. Scale bar: 100µm. A': EGFP fluorescence quantification in the TeO of m*Stat3C* injected and control larvae at 48 hpf. Statistical analysis was performed by unpaired t-test (n=20); **p<0.01 B: representative pictures of 24-hpf *Tg(7xStat3:EGFP)* injected with stat3-MO1 (Liu *et al.,* 2017; Miyagi *et al.,* 2004; Yamashita *et al.,* 2002) 5-mismatch morpholino B': Quantification of EGFP fluorescence in 24-hpf embryos injected with stat3-MO1 morpholino and 5-mismatch morpholino on the reporter line. Scale bar=250µm, statistical analysis was performed by unpaired t-test on 3 biological replicates. **p<0.01, ****p<0.0001; error bars=SEM. C: dorsal view of 48-hpf *Tg(7xStat3:EGFP)* injected with stat3-MO1

Development • Supplementary information

and 5-mismatch morpholino. C’: Quantification of EGFP fluorescence in 48-hpf *Tg(7xStat3:EGFP)* embryos injected with stat3-MO1 morpholino and 5-mismatch morpholino. *p<0.05, **p<0.01; error bars=SEM

Development • Supplementary information

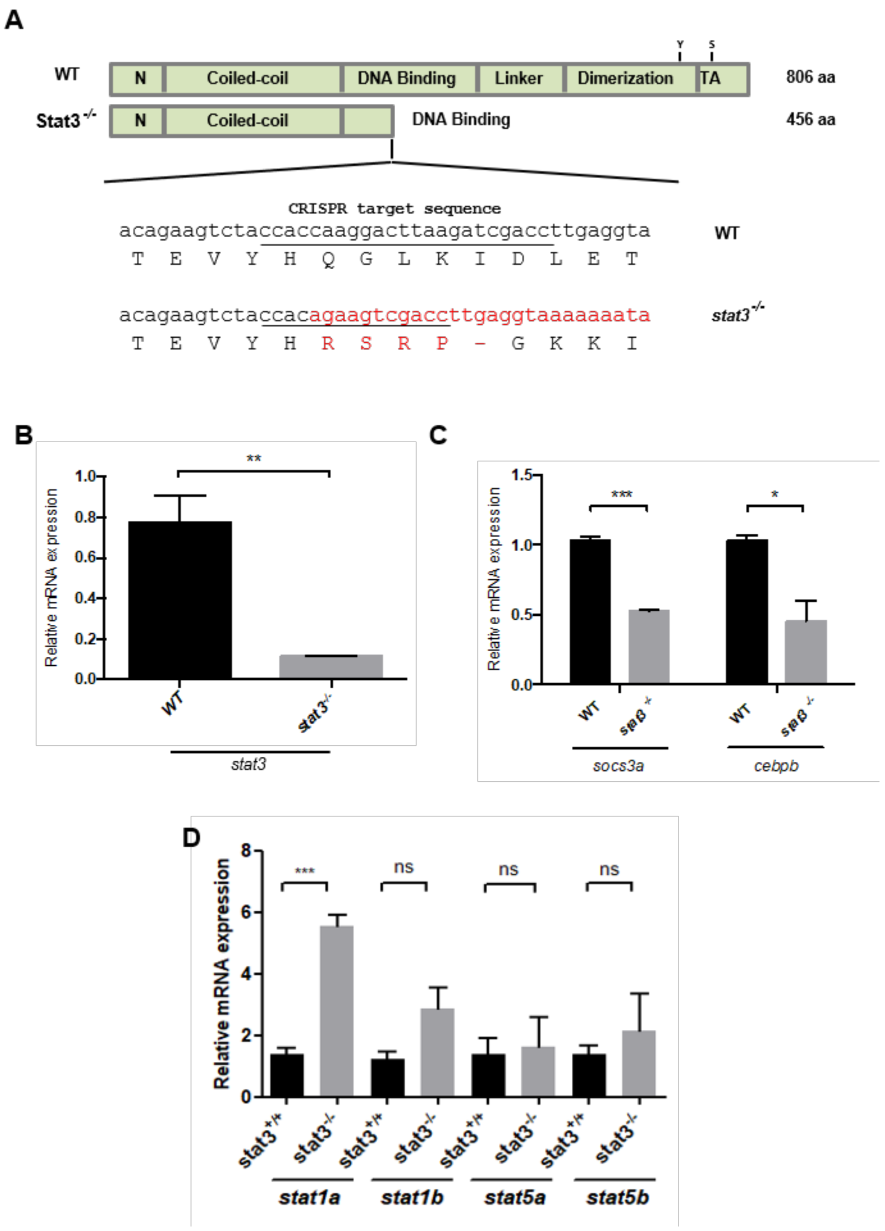


**Fig. S4: *stat3$^{ia23}$* mutant validation.** A: Schematic representation of *stat3$^{-/-}$* mutant allele in comparison with WT. B: qRT-PCR analysis of *stat3* mRNA expression normalized on *gapdh* from WT and *stat3$^{-/-}$* siblings at 6 dpf (p-value= 0,0073). C: qRT-PCR analysis of *socs3a* and *cebpb* Stat3 targets expression normalized on *gapdh* from WT and *stat3$^{-/-}$* siblings at 6 dpf, *zgapdh* was used as internal control (p-values= 0,001; 0,0205); Statistical analysis was performed by unpaired t-test on 3 independent biological samples. **p<0,01; error bars=SEM. D: qRT-PCR analysis of *stat1a*, *stat1b*, *stat5a* and *stat5b* mRNA expression levels normalized on *gapdh* from *stat3$^{+/+}$* and *stat3$^{-/-}$* siblings at 6 dpf (***p<0.001)

Development • Supplementary information

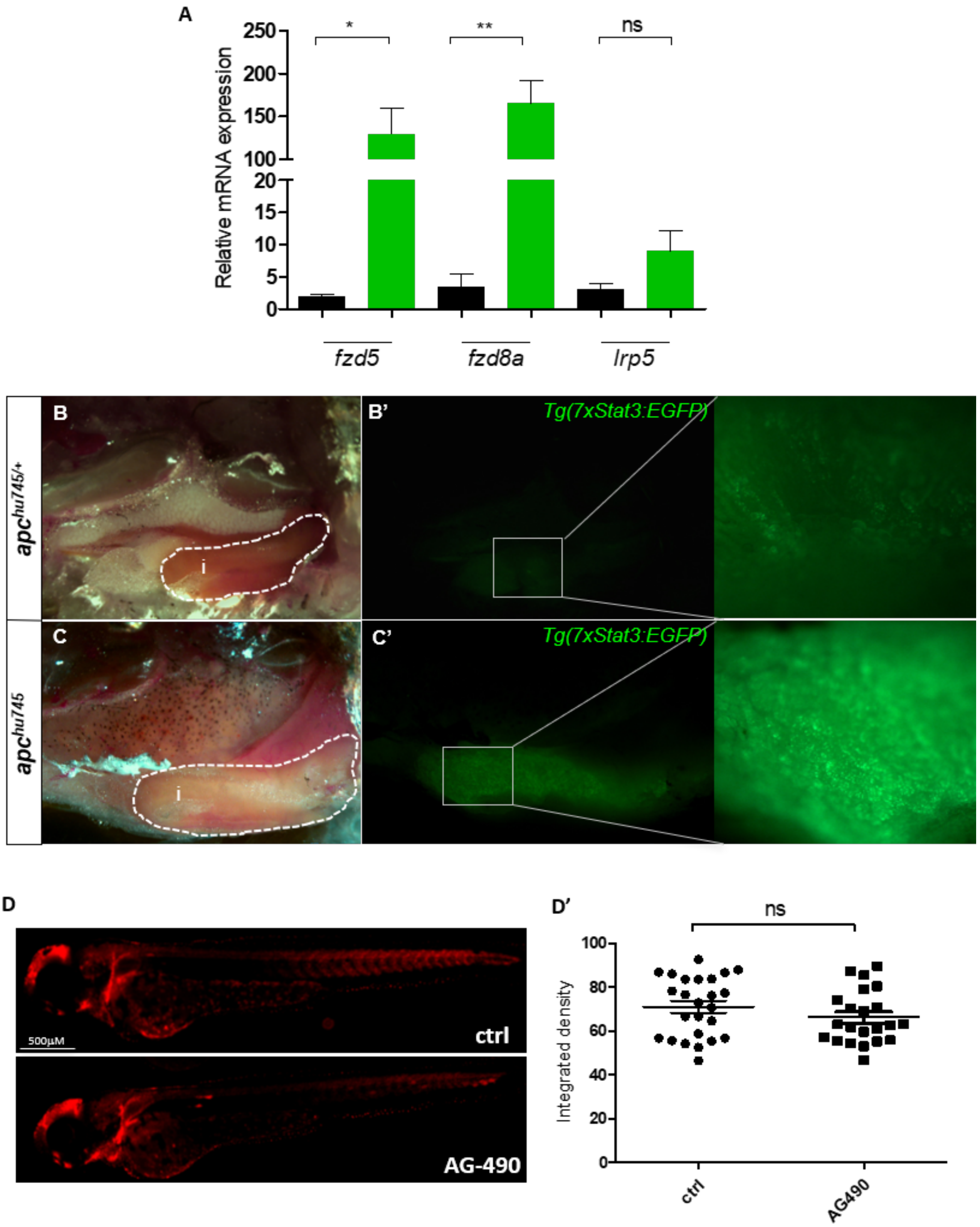


**Fig. S5: Stat3 pathway is strongly activated in zebrafish *apc*-driven tumor.** A: qRT-PCR analysis of *fzd5*, *fzd8a* and *lrp5* on EGFP-positive and EGFP-negative cells sorted from adult intestines. B-C'': *In vivo* EGFP expression is ectopic in 12 mpf *Tg(7xStat3:EGFP)/*$apc^{hu745}$ hyperplastic intestine (B-B') with respect to *Tg(7xStat3:EGFP)/* $apc^{hu745/+}$ siblings (B-B'). C-C': representative pictures of 3-dpf

Development • Supplementary information

*Tg(7xTCF-Xla.Siam:nlsmCherry)*$^{ia5}$ reporter larvae treated with DMSO and 80 µM AG-490 from 8-72 hpf (D); quantification of *Tg(7xTCF-Xla.Siam:nlsmCherry)*$^{ia5}$ reporter larvae fluorescence (D'). Statistical analysis was performed by unpaired t-test. ns=not significant.

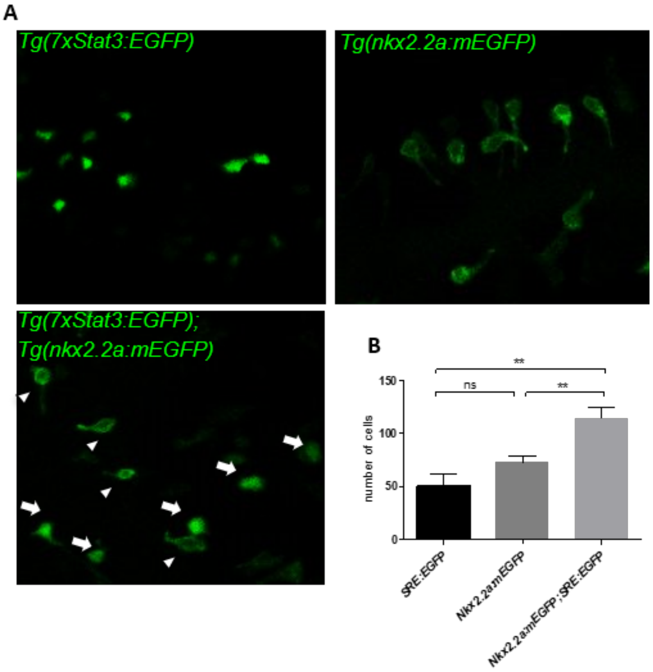


**Fig. S6: EGFP positive cells of *Tg(7xStat3:EGFP)* zebrafish line are not secretory cells.** A: representative prictures of *Tg(7xStat3:EGFP)*, *Tg(nkx2.2a:mEGFP)* and *Tg(7xStat3:EGFP)/Tg(nkx2.2a:mEGFP)* 6-dpf larvae intestine. B: number of EGFP-positive cells measured in intestines of *Tg(7xStat3:EGFP)*, *Tg(nkx2.2a:mEGFP)* and *Tg(7xStat3:EGFP)/Tg(nkx2.2a:mEGFP)* 6-dpf. Arrows indicate *Tg(nkx2.2a:mEGFP)* positive cells; arrowheads indicate *Tg(7xStat3:EGFP)* positive cells. **p<0.01, ns=not significant; error bars=SEM.

Development • Supplementary information

| Gene | Forward primer sequence | Reverse primer sequence |
|---|---|---|
| *zstat3* | TGCCACCAACATCCTAGTGT | GCTTGTTTGCACTTTTGACTGA |
| *zgapdh* | GTGGAGTCTACTGGTGTCTTC | GTGCAGGAGGCATTGCTTACA |
| *zcebpb* | CCAAAAGTAACGGGCGACAC | ATCTTCCCTTACCTGACGGC |
| *zsocs3* | GGAAGACAAGAGCCGAGACT | GCGATACACACCAAACCCTG |
| *egfp* | ACGTAAACGGCCACAAGTTC | AAGTCGTGCTGCTTCATGTG |
| *zstat1a* | GCAGCTCAAGAAACTCCTGG | AAAGGTCTCTGCAGTTGGGT |
| *zstat1b* | CGAGTGGAAGAAGAGACAGC | GCTGGCCCCTTCCTAGATTT |
| *zstat5a* | TGACCCGAGAAGCTAACACC | GTATGTCCAGTCCTCCCT |
| *zstat5b* | TGAGGAAACAGCAAACCGTG | GCTGCTGAGTCAAGTGTTCA |
| *zil6* | CGTAAAGAGTCTCCTTGGCG | GGTTTGAGGAGAGGAGTGCT |
| *zgp130* | TGCTGGAGTGGGTGAATGAA | GGCTTGGTTACTGGTGTTCC |
| *zjak2a* | CTTCGAGAGTCAGGAGCCC | CTGAAGCTTCTTCACCGCC |
| *zjak2b* | ACGTATTGTGATTTCGCGGA | ACAAAAGACAAGGCCTGCAT |
| *zsox9b* | CTCGGCAAACTCTGGAGACT | GCGCATTGGTGGAGATCTG |
| *zagr2* | GCACAGACATACGAGGAAGC | GGAGACAAGTGCTTATCTGTG |
| *zpept1* | GATTGCTTTGGGAACAGGAGG | GATGGGTGTGATGAGAGTGG |
| *zfabp2* | GCTGCCCATGACAACCTG | CGTGTCTCCCTCTATGACC |
| *znotch2* | GACGAATGCATCTCCAGTGC | GCAGCAGCCACAGCAACC |
| *zpcna* | CCTTGGCACTGGTCTTTGAA | GGCACACGAGATCATGACAG |
| *zcyclinD1* | CCAACTTCCTCTCGCAAGTC | TGGTCTCTGTGGAGATGTGC |
| *zfzd5* | CCTAACTGTGCACTGCCTTG | ATTTGAAGCGCTCCATGTCG |
| *zfzd8a* | TGCAATCGGGAGTATGACGT | CTCGTTTCCCCACTTCATGC |
| *zlrp5* | TTCTCGGAGGGCCTGATTTT | TTGTCTCCGAGTCAGTCCAG |

**Tab. 1:** list of primer used for Real Time qPCR.

Development • Supplementary information
